# Statistical Analysis Methods and Reporting of Patient-Reported Outcomes in Randomized Controlled Trials for Cancer Conducted in Japan: A Systematic Review

**DOI:** 10.7759/cureus.60804

**Published:** 2024-05-21

**Authors:** Junki Mizusawa, Gakuto Ogawa, Mitsumi Terada, Hiroto Ishiki, Yuichiro Kikawa, Naomi Kiyota

**Affiliations:** 1 Center for Research Administration and Support, National Cancer Center, Tokyo, JPN; 2 Department of International Clinical Development, National Cancer Center Hospital, Tokyo, JPN; 3 Department of Palliative Medicine, National Cancer Center Hospital, Tokyo, JPN; 4 Department of Breast Surgery, Kansai Medical University, Osaka, JPN; 5 Department of Medical Oncology and Hematology, Cancer Center, Kobe University Hospital, Kobe, JPN

**Keywords:** health-related quality of life (hrqol), randomized trials, pro, hrqol, randomized controlled trial, statistical methods, health-related quality of life, patient reported outcomes

## Abstract

The Setting International Standards in Analyzing Patient-Reported Outcomes and Quality of Life Endpoints Data (SISAQOL) initiative was established in 2016 to assess the quality and standardization of patient-reported outcomes (PRO) data analysis in randomized controlled trials (RCTs) on advanced breast cancer. The initiative identified deficiencies in PRO data reporting, including nonstandardized methods for handling missing data. This study evaluated the reporting of health-related quality of life (HRQOL) in Japanese cancer RCTs to provide insights into the state of PRO reporting in Japan. The study reviewed PubMed articles published from 2010 to 2018. Eligible studies included Japanese cancer RCTs with ≥50 adult patients (≥50% were Japanese) with solid tumors receiving anticancer treatments. The evaluation criteria included clarity of the HRQOL hypotheses, multiplicity testing, primary analysis methods, and reporting of clinically meaningful differences. Twenty-seven HRQOL trials were identified. Only 15% provided a clear HRQOL hypothesis, and 63% examined multiple HRQOL domains without adjusting for multiplicity. Model-based methods were the most common statistical methods for the primary HRQOL analysis. Only 22% of the trials explicitly reported clinically meaningful differences in HRQOL. Baseline assessments were reported in most trials, but only 26% reported comparisons between the treatment groups. HRQOL analysis was based on the intention-to-treat population in 19% of the trials, and 74% reported compliance at follow-up; however, 41% did not specify how missing values were handled. Although the rates of reporting clinical hypotheses and clinically meaningful differences were relatively low, the current state of HRQOL evaluation in the Japanese cancer RCT appears comparable to that of previous studies.

## Introduction and background

In recent years, the importance of patient-reported outcomes (PRO) for health-related quality of life (HRQOL) has been increasing [1] in addition to conventional objective endpoints such as overall survival and response rate in the field of oncology. Although multiple guidelines have been proposed for reporting results [2-4], the use of HRQOL/PRO is not fully established owing to its ambiguity, various clinical hypotheses, and complex statistical methods. Between 2010 and 2020, only 8.3% and 30.2% of drugs approved by the FDA and the European Medicines Agency, respectively, in the oncology field were approved with PRO labeling, indicating that PRO has not yet been widely adopted in a form that can withstand evaluation by strict regulatory agencies such as the FDA [5].

In 2016, the Setting International Standards in Analyzing Patient-Reported Outcomes and Quality of Life Endpoints Data (SISAQOL) initiative was established to provide recommendations for standardizing the analysis of HRQOL and other PRO data in randomized controlled trials (RCT) regarding cancer [6]. In addition to the work of the FDA [7], the SISAQOL Consortium performed a systematic review to assess the variability, quality, and standards of PRO data analysis in RCTs on advanced breast cancer. The study findings showed a poor current situation in the reporting of PRO data and that the methods of analysis and handling of missing data have not been standardized [8].

Japanese researchers have also recognized the value of HRQOL/PRO as a critical endpoint in cancer-related clinical trials. In the 2021 revised guidelines for the clinical evaluation of anticancer drugs, the importance of PRO was explicitly stated for the first time [9], and the Japan Clinical Oncology Group (JCOG), one of the largest investigator-led cooperative groups in Japan, established a PRO/QOL research committee and published its policies [10]. However, no survey studies on HRQOL/PRO, such as those conducted by the SISAQOL, have been conducted for RCTs involving cancer in Japan, and it is unclear whether the current state of HRQOL/PRO in Japan is comparable to that in Europe and the United States.

This study aimed to evaluate reports of Japanese cancer RCTs that utilized PRO/QOL using the same criteria as the previous SISAQOL study to determine potential differences in the statistical analysis methods used between Japan and other countries and whether any specific issues are unique to Japanese trials.

## Review

Methods

We used the methodology described in the Cochrane Handbook for Systematic Reviews of Interventions, and the results of this systematic review are reported according to the Preferred Reporting Items for Systematic Reviews and Meta-Analyses (PRISMA) guidelines [11]. We conducted a literature search of PubMed on April 9, 2019 using the following keywords: (quality of life[MeSH Terms] OR quality of life[Text Word]) AND cancer[Text Word] AND (Japan [All Fields]) AND (Randomized Controlled Trial) AND (neoplasm[MeSH Terms]) AND (Clinical Trial[ptyp] AND (“2010/01/01” [PDat]: “2018/03/30” [PDat]) AND Humans[Mesh]). Using this search strategy, we identified 125 potentially eligible articles written in English and reviewed the references of these publications for additional articles. We also performed a Web of Science search on April 25, 2019 and found one article.

The inclusion and exclusion criteria for the RCTs were similar to those reported by Pe et al. [8]. The inclusion criteria were the following: (1) RCT articles published between 2010 and 2018; (2) reporting of PRO findings; (3) a study population of adult patients (≥18 years of age) with solid tumor cancer receiving anticancer treatments (chemotherapy, targeted therapy, endocrine therapy, immunotherapy, surgery, radiotherapy, and endoscopy); (4) a sample size of at least 50 patients; and (5) Japanese persons comprising 50% of the enrolled patients (essentially, patients are enrolled for clinical trials from institutions within Japan.).

**Figure 1:**
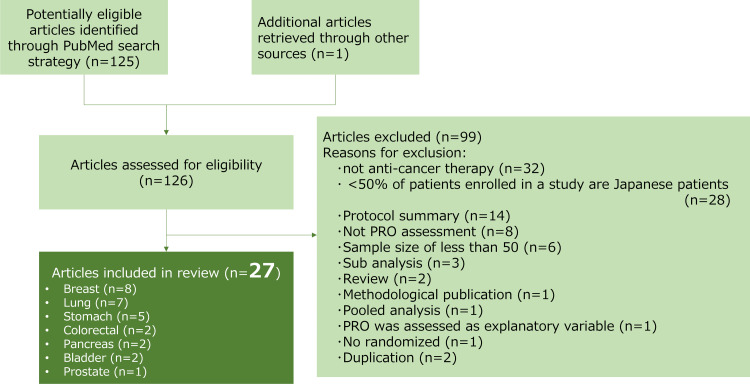
Study flowchart

We excluded all RCTs that evaluated psychological, supportive, or supplementary interventions. Supplementary treatment was defined as an intervention other than anticancer therapies. We excluded purely methodological publications and review reports. We did not consider quality-adjusted life-year endpoints as PRO endpoints. Publications reporting interim analyses or analyses of patient subgroups were also excluded. Figure 1 shows a flowchart of the search strategy and the inclusion and exclusion criteria. JM and MT independently reviewed 126 eligible studies and assessed whether the reports met the inclusion and exclusion criteria. Any disagreements between them in the study assessments were discussed and resolved.

JM and GO independently extracted the information using predefined data abstraction forms. All data were checked for internal consistency, and disagreements were resolved by discussion. The following details were extracted: a general description of the article, research objectives, statistical analysis and clinical relevance, baseline assessment, and assessment of the amount and handling of missing data. The extracted information was summarized.

Results

Study Selection

Of the 126 eligible papers, 27 were selected for the systematic review [12-38]. General information and summary results of the 27 articles are shown in Table 1. Eight studies focused on breast cancer, seven on lung cancer, and five on stomach cancer; the other papers focused on colorectal, pancreatic, bladder, and prostate cancers. The details of the classification of the selected 27 trials are presented in Table 2.

**Table 1:** General information and summary of the 27 articles included in the study

	Yes	No	Not reported or unclear
Reporting of research objectives			
Specific hypothesis	4	10	13
Statistical significance and clinical relevance			
Multiple domains	17	10	0
If yes, was statistical correction used?	0	3	14
Repeated assessments	24	3	0
If yes, was a statistical technique used that allowed the inclusion of repeated assessment points, or was a statistical correction used?	16	7	1
Reporting of descriptive data	17	10	0
Primary statistical method			
Linear mixed models	7	NA	NA
Wilcoxon rank-sum test or t-test	6	NA	NA
ANOVA or linear regression	2	NA	NA
Time to event	2	NA	NA
Repeated measures ANOVA	2	NA	NA
Proportion of patients or responder analysis	1	NA	NA
Others	3	NA	NA
Unreported or unclear	4	NA	NA
Reporting of clinical relevance	6	21	0
Change of X points from baseline	4	NA	NA
X points difference between arms	1	NA	NA
Change of X points from baseline and X points difference between arms	1	NA	NA
Baseline assessment			
Assessed baseline	24	2	1
Compared baseline scores between treatments	7	17	0
Included baseline as a covariate	12	11	1
Assessing the prevalence and handling of missing data			
Intention-to-treat population	5	18	4
Baseline compliance rates for each treatment arm	16	11	NA
Follow-up compliance rates for each treatment arm	20	7	NA
Strategy to handle missing data	16	11	NA

**Table 2:** Details of the classification of the selected 27 trials

Author	Year	Cancer type	Disease status	Specific hypothesis	Multiple domains (more than one scale or domain included in the analysis)	QOL questionnaire	If yes, was statistical correction used (multiple domains were independently tested)?	Repeated assessments (more than one follow-up assessment included in the analysis)	If yes, was a statistical technique used that allowed the inclusion of repeated assessment points, or was a statistical correction used (if repeated assessments were independently tested)?	Reporting of descriptive data	Primary statistical technique	Not reported or unclear	(Generalized) linear mixed models, including pattern mixture models	Wilcoxon rank-sums test or between-subjects t-test	ANOVA or linear regression	Time to event	Repeated measures ANOVA	Proportion of patients or responder analysis	Others	Reporting of clinical relevance	Change of X points (from baseline)	X points difference (between arms)	Change of X points from baseline and X points differences (between arms)	Assessed baseline	Compared baseline scores between treatment arms	Included baseline as a covariate	Intention-to-treat population	Baseline compliance rates for each treatment arm	Follow-up compliance rates for each treatment arm	Strategy to handle missing data
Hagiwara, Y	2018	Pancreas	Curative	Yes	No	EQ-5D-3L		Yes	Yes	Yes	Yes	No	Yes	No	No	No	No	No	No	Yes	Yes	No	No	Yes	Yes	Yes	No	Yes	Yes	Yes
Kawahara, T	2018	Breast	Unresectable	No	Yes	EORTC QLQ-C30, Patient Neurotoxicity Questionnaire	No	Yes	Yes	Yes	Yes	No	No	No	No	Yes	No	No	No	Yes	Yes	No	No	Yes	No	No	No	No	Yes	Yes
Ohashi et, Y	2018	Breast	Curative	No	Yes	QOL-ACD, QOL-ACD-B, FACT-ES	No	Yes	Yes	Yes	Yes	No	Yes	No	No	No	No	No	No	No	No	No	No	Yes	Yes	Yes	Yes	No	No	Yes
Yamamoto, D	2017	Breast	Unresectable	No	Yes	EORTC-QLQ-C30	No	Yes	No	No	No	Yes	No	No	No	No	No	No	No	No	No	No	No	Yes	No	No	No	Yes	Yes	No
Shiroiwa, T	2017	Breast	Unresectable	No	No	EQ-5D		Yes	Yes	Yes	Yes	No	Yes	No	No	No	No	No	No	Yes	Yes	No	No	Yes	Yes	Yes	No	Yes	Yes	Yes
Yoshino, S	2016	Gastric	Unresectable	No	No	FACT-Biological Response Modifier		Yes	No	No	No	Yes	No	No	No	No	No	No	No	No	No	No	No	Yes	No	No	No	Yes	No	No
Yamazaki, K	2016	Colorectal	Unresectable	No	Yes	FACT-C, FACT/GOG-Ntx	Not reported or unclear	Yes	Yes	No	Yes	No	No	No	No	No	Yes	No	No	No	No	No	No	Yes	No	No	Not reported or unclear	No	No	Yes
Nakamura, M	2016	Gastric	Curative	Yes	Yes	FACT-Ga, FACT-G	Not reported or unclear	Yes	No	Yes	No	Yes	No	No	No	No	No	No	No	Yes	No	No	Yes	Yes	No	No	Yes	Yes	Yes	Yes
Yokomizo, A	2016	Bladder	Curative	Not reported or unclear	Yes	EORTC QLQ-C30	Not reported or unclear	No		Yes	Yes	No	No	Yes	No	No	No	No	No	No	No	No	No	Yes	No	No	No	Yes	Yes	No
Ito, Y	2016	Gastric	Curative	Yes	Yes	EORTC-QLQ-C30, STO22	Not reported or unclear	Yes	No	No	Yes	No	No	Yes	No	No	No	No	No	Yes	No	Yes	No	Yes	No	No	No	Yes	Yes	Yes
Kubota, K	2015	NSCLC	Unresectable	Not reported or unclear	Yes	EORTC-QLQ-C30, QLQ-LC13	Not reported or unclear	Yes	Yes	No	Yes	No	No	No	No	No	Yes	No	No	No	No	No	No	Yes	No	Yes	Not reported or unclear	No	Yes	No
Masuda, K	2015	Rectal	Curative	Not reported or unclear	No	Fecal Incontinence Quality of Life		Yes	No	Yes	Yes	No	No	Yes	No	No	No	No	No	No	No	No	No	Not reported or unclear			No	No	No	No
Abe, T	2015	NSCLC	Unresectable	Not reported or unclear	No	FACT-L		Yes	Yes	No	Yes	No	No	No	No	No	No	Yes	No	No	No	No	No	Yes	No	Yes	No	Yes	Yes	Yes
Matsuyama, H	2015	Prostate	Curative	No	Yes	Expanded Prostate Cancer Index Composite	Not reported or unclear	Yes	Not reported or unclear	No	No	Yes	No	No	No	No	No	No	No	No	No	No	No	Yes	Yes	No	No	No	No	No
Tsukada, H	2014	NSCLC	Unresectable	Not reported or unclear	No	FACT-L		No		Yes	Yes	No	No	No	Yes	No	No	No	No	No	No	No	No	Yes	No	Yes	Yes	Yes	Yes	No
Sekine, I	2013	SCLC	Unresectable	Not reported or unclear	Yes	FACT-L, EQ-5D	Not reported or unclear	Yes	Yes	No	Yes	No	No	No	Yes	No	No	No	No	No	No	No	No	Yes	No	Yes	No	Yes	Yes	No
Ueno, H	2013	Pancreas	Unresectable	No	No	EQ-5D		Yes	Yes	No	Yes	No	No	No	No	No	No	No	Yes	No	No	No	No	Yes	No	No	Not reported or unclear	No	No	Yes
Shimozuma, K	2012	Breast	Curative	Yes	Yes	Patient Neurotoxicity Questionnaire, FACT-G, FACT-Neuro-toxicity	Not reported or unclear	Yes	Yes	Yes	Yes	No	No	Yes	No	No	No	No	No	No	No	No	No	Yes	No	Yes	No	Yes	Yes	Yes
Oizumi, S	2012	NSCLC	Unresectable	Not reported or unclear	No	Care Notebook		Yes	Yes	Yes	Yes	No	No	No	No	Yes	No	No	No	Yes	Yes	No	No	Yes	Yes	No	No	Yes	Yes	No
Takei, H	2012	Breast	Curative	Not reported or unclear	Yes	FACT-B, FACT-ES, CES-D	Not reported or unclear	Yes	Yes	No	Yes	No	Yes	No	No	No	No	No	No	No	No	No	No	Yes	No	Yes	No	Yes	Yes	Yes
Takiguchi, S	2012	Gastric	Curative	Not reported or unclear	Yes	EORTC-QLQ-C30, DAUGS 20	Not reported or unclear	No		Yes	Yes	No	No	Yes	No	No	No	No	No	No	No	No	No	No			No	No	Yes	Yes
Ishigami, S	2011	Gastric	Curative	No	No	Original		Yes	No	Yes	Yes	No	No	Yes	No	No	No	No	No	No	No	No	No	No			No	No	Yes	No
Kawahara, M	2011	NSCLC	Unresectable	Not reported or unclear	Yes	FACT-L, FACT-Taxane, FACIT-Sp	Not reported or unclear	Yes	Yes	Yes	Yes	No	Yes	No	No	No	No	No	No	No	No	No	No	Yes	Yes	Yes	No	Yes	Yes	Yes
Shiroiwa, T	2011	Breast	Curative	No	Yes	EQ-5D, FACT-G, FACT-B, FACT-Taxane	Not reported or unclear	Yes	Yes	Yes	Yes	No	Yes	No	No	No	No	No	No	No	No	No	No	Yes	No	Yes	Yes	No	Yes	Yes
Ohsumi, S	2011	Breast	Curative	Not reported or unclear	Yes	FACT-B, FACT-ES, CES-D	Not reported or unclear	Yes	Yes	Yes	Yes	No	No	No	No	No	No	No	Yes	No	No	No	No	Yes	Yes	Yes	No	Yes	Yes	Yes
Koga, H	2010	Bladder	Curative	Not reported or unclear	Yes	EORTC-QLQ-C30	Not reported or unclear	Yes	No	Yes	Yes	No	No	No	No	No	No	No	Yes	No	No	No	No	Yes	No	No	Not reported or unclear	No	No	No
Takeda, K	2010	NSCLC	Unresectable	Not reported or unclear	No	FACT-L		Yes	Yes	Yes	Yes	No	Yes	No	No	No	No	No	No	No	No	No	No	Yes	No	Not reported or unclear	Yes	Yes	Yes	Yes

HRQOL Measurement

Several HRQOL questionnaires were used: the EORTC-QLQ-C30 [39] was most commonly used (seven studies, 26%), followed by the EQ-5D (five studies, 19%); EORTC-developed disease-specific questionnaires for gastric cancer, EORTC-QLQ-STO 22 [40,41], and the EORTC QLQ-LC13 [42] for lung cancer were used in one study each. The Functional Assessment of Cancer Therapy (FACT) questionnaire was widely used, including FACT-L for lung cancer [43], FACT-B for breast cancer [44], FACT-General for general cancer [45], and FACT-ES for endocrine therapy [46]. The Quality of Life Questionnaire for Patients treated with Anticancer Drugs (QOL-ACD) [47] and the QOL-ACD-B (breast) questionnaires developed in Japan were used in one study each. The Patient Neurotoxicity Questionnaire, a questionnaire for specific adverse events, was also used in two studies.

Reporting of Research Objectives

First, we examined whether a predefined hypothesis regarding HRQOL was stated. Only four studies (15%) were judged to have a predefined statement that noted a specific PRO domain and time point or time frame, 10 studies (37%) had statements that were considered unclear (for example, “to explore the relationships between the QOL”), and 13 studies (48%) had no statement.

Multiplicity Adjustment

To investigate the issue of multiplicity in testing, we assessed whether multiple domains of HRQOL were examined. Our findings revealed that of the 27 trials, 17 (63%) examined more than one HRQOL domain. However, none of these trials made clear adjustments for multiplicity in their analysis of multiple domains, and 14 provided an unclear description of such adjustments. In addition, HRQOL was assessed at multiple time points in 24 trials (89%), of which 16 (59%) described the multiplicity adjustment. Seven trials (26%) did not describe these adjustments.

Statistical Analysis and Clinical Relevance

Descriptive statistics for HRQOL were reported in 17 trials (63%). Various statistical methods were used for the primary HRQOL analysis. The most commonly used method was the linear mixed model, which was used in seven trials (26%). Model-based methods accounted for 11 trials (41%) in all analyses when ANOVA and repeated-measures ANOVA were included. Wilcoxon rank-sum tests and t-tests were used in six trials (22%) to compare simple means or medians. Two trials used time-to-event methods, whereas one trial used tests of proportions, such as the chi-square or Fisher’s exact test. Four trials did not report the primary analysis method.

Only six trials (22%) explicitly specified and reported clinically meaningful differences in HRQOL. Among these, four defined a clinically meaningful difference as a change from baseline, one trial defined it as a difference between treatment groups, and one trial used a combination of both definitions.

Of the 27 trials, baseline assessments were reported in 24 (89%), one (4%) was unclear, and two (7%) did not report baseline assessments. Among the 24 trials with baseline assessments, only seven reported comparisons between the treatment groups. However, 12 trials used baseline values as covariates in their statistical analyses.

The HRQOL analysis was based on the intention-to-treat population in five trials (19%) and the modified intention-to-treat population, which is defined as a population, not including all randomized patients. However, some patients were excluded from the entire population in 18 trials (67%), while four trials had unclear descriptions of the analysis population. Compliance rates were evaluated to determine the amount of missing data. Baseline compliance for the HRQOL assessment was reported in 16 trials (59%), and compliance at follow-up was reported in 20 trials (74%). Additionally, 11 trials (41%) did not report how the missing values were handled.

Discussion

This systematic review of 27 randomized clinical trials for anticancer treatment evaluated HRQOL in Japanese cancer patients, including the clarity of the HRQOL hypothesis, correction for multiplicity testing, primary analysis methods used, reporting of clinically meaningful differences, and reporting of missing data. Only 15% of the trials had a clear statement about the HRQOL-predefined hypothesis, and 63% examined more than one HRQOL domain without explicit adjustments for multiplicity. Various statistical methods were used for the primary HRQOL analysis, with model-based methods being the most common. Only 22% of the trials explicitly reported clinically meaningful differences in HRQOL, and baseline assessments were reported in most trials; however, only 26% reported comparisons between treatment groups. The HRQOL analysis was based on the intention-to-treat population in 19% of the trials, and compliance at follow-up was reported in 74% of the trials; however, 41% did not report how missing values were handled.

Our study aimed to compare the proportion of essential contents and the differences in statistical methods used in Japanese cancer RCT PRO/QOL papers with those in a study on unresectable/metastatic breast cancer evaluated by SISAQOL and to examine whether there are any unique issues specific to Japan. Regarding the presence of a specific hypothesis, only 12% of the articles in the SISAQOL study reported a specific hypothesis [8], with similarly low values in our study. This may be because HRQOL endpoints have many variations in hypotheses compared to the usual endpoints of cancer, such as overall survival or response rate, and HRQOL itself is often positioned as an exploratory secondary endpoint in clinical trials; therefore, many research plans may not have a clear hypothesis in advance [48]. Although guidelines such as ISOQOL [2] and CONSORT-PRO [3] require a clear description of hypotheses regarding HRQOL, it is impossible to describe them in a paper if they are not outlined in the research plan. Recently, an extended version of the SPIRIT guidelines, which specify the items to be included in research plans, was proposed for PRO research to provide evidence-based recommendations for the minimum content of a clinical trial protocol [49]. To improve the low rate of hypothesis description regarding HRQOL, these guidelines should be widely disseminated for clear hypotheses regarding HRQOL to be established from the research planning stage. In Japan, the JCOG has established and published policies regarding PRO and QOL research [10]. Such endeavors are crucial and are expected to remain significant in the future.

Various statistical methods were used for the primary statistical analysis, including model-based methods, the Wilcoxon rank-sum test, and the t-test, similar to those used in the previous SISAQOL study. In the SISAQOL study, model-based methods were used in 44% of studies, whereas the Wilcoxon rank-sum test and t-test were used in 17%. Appropriate statistical methods should be selected based on the clinical hypotheses and outcome types. If the method is chosen accordingly, it is considered sufficient. The SISAQOL-IMI consortium recommends statistical methods based on the outcome type and clinical hypotheses, which are useful for discussions between biostatisticians and clinicians [50]. However, no consensus has been reached for some clinical hypotheses, such as comparing QOL scores over time. A recent publication has reported details of recommendations regarding the views and opinions of PRO objectives and endpoints for RCTs from 41 stakeholders [51].

In the previous SISAQOL study, 42% of patients reported minimally important differences (MIDs), which is a measure of clinical relevance, whereas the reported rate was 22% in the present study. This may be related to the lower percentage of stated clinical hypotheses regarding HRQOL because, to clearly define a clinical hypothesis, its MIDs must be determined. Simultaneously, in cancer clinical trials, the sample size required to detect the primary endpoint, overall survival, or progression-free survival is usually larger than that required to detect an MID in HRQOL. Therefore, whether an MID was achieved is more important than whether a statistically significant difference was achieved [52]. MID has other challenges, as it can vary depending on the cancer type and domain. However, methods for defining MID are being established, and such efforts may contribute to the adoption and widespread use of MID, along with the prior establishment of its definition [53].

The reported rate of adjustment for multiplicity in statistical hypothesis testing was approximately 60%, similar to that of the SISAQOL study but not sufficiently high. At the very least, a prespecified adjustment for multiplicity is required for a drug to be accepted by regulatory agencies, such as the FDA, and listed on the drug label. In fact, in a review of FDA-approved drugs in clinical trials for breast cancer, the FDA reviewer’s comments suggested that, in addition to the lack of MIDs, inadequate analytical methods due to uncontrolled multiple comparisons may be the reason for the lack of drug product labeling [54].

The number of articles describing the handling of missing data and compliance rates tended to be higher than in the previous SISAQOL study. This finding may be partly because several of the Japanese clinical trials were sub-papers of RCTs limited to HRQOL/PRO endpoints, and the first author was a biostatistician. It is difficult to determine whether this is an appropriate procedure in cancer clinical trials, where there is much missing data and HRQOL often includes deaths. The ICH E9(R1) guidelines provide an estimand framework (treatment, population, variables, population-level summary, and handling of intercurrent events) for defining the treatment effect under investigation in a clinical trial [55]. The concept of estimands for HRQOL in cancer clinical trials has been proposed [56,57], and it is hoped that this and future studies will help to build a consensus.

Limitations

This study had some limitations. The findings were restricted to RCTs published between 2010 and 2018 in English and cannot be generalized to other published years and languages. Although the previous review by SISAQOL limited to advanced breast cancer [8], this study was not restricted to breast cancer to increase the number of publications. One limitation is the inability to compare with studies conducted under frameworks other than the SISAQOL project [58]. Although there were no notable differences between breast cancer and other cancer types, it is important to note that if there are differences based on the cancer type, descriptions regarding comparisons may not always be accurate. Furthermore, the description of HRQOL, a secondary endpoint, may have been omitted in papers with word count limits. In RCTs where HRQOL/PRO is evaluated as secondary endpoints, there is a tendency for HRQOL/PRO to be reported as separate papers, referred to as secondary papers. While such studies were limited in this review, it is expected that independent papers on HRQOL/PRO will contain a wealth of information.

## Conclusions

Although the reporting rates of clinical hypotheses and MIDs in the previous reports tended to be similar to those in our study, the reporting rates for HRQOL/PRO compliance and the handling of missing values tended to be higher in previous reports. Overall, the statistical methods used for HRQOL/PRO evaluation in the Japanese cancer RCT were similar to those used in the previous SISAQOL study, indicating that the reporting methods of Japanese studies are not inferior to those of Western countries. The standardization of statistical and reporting methods is expected to progress domestically and internationally, following the guidelines presented by SISAQOL and regulatory agencies.
